# Improving paramedic responses for patients dying at home: a theory of change-based approach

**DOI:** 10.1186/s12873-023-00848-0

**Published:** 2023-08-02

**Authors:** Jane Simpson, Bader Nael Remawi, Kieran Potts, Tania Blackmore, Maddy French, Karen Haydock, Richard Peters, Michael Hill, Oliver-Jon Tidball, Georgina Parker, Michelle Waddington, Nancy Preston

**Affiliations:** 1 Division of Health Research, Lancaster University, Lancaster, LA1 4YT UK; 2 Lancaster Medical School, Lancaster University, Lancaster, LA1 4YT UK; 3 North West Ambulance Service NHS Trust, Bolton, BL1 5DD UK; 4 Heart of Kent Hospice, Preston Hall, Aylesford, Kent, ME20 7PU UK

**Keywords:** Paramedic, End-of-life care, End of life, Theory of change, Terminal care, Decision-making, Workshops

## Abstract

**Background:**

Paramedics are increasingly being called to attend patients dying from advanced incurable conditions. However, confidence to deal with such calls varies, with many feeling relatively unskilled in this aspect of their role. A number of interventions have been piloted to improve their skills in end-of-life care (EoLC) but without a fully specified theoretical model. Theory of Change models can provide theoretical and testable links from intervention activities to proposed long-term outcomes and indicate the areas for assessment of effectiveness. This study aimed to develop an intervention for improving paramedic EoLC for patients in the community.

**Methods:**

A Theory of Change approach was used as the overarching theoretical framework for developing an intervention to improve paramedic end-of-life skills. Nine stakeholders - including specialist community paramedics, ambulance call handlers and palliative care specialists - were recruited to five consecutive online workshops, ranging between 60 and 90 min. Each workshop had 2–3 facilitators. Over multiple workshops, stakeholders decided on the desired impact, short- and long-term outcomes, and possible interventions. During and between these workshops a Theory of Change model was created, with the components shared with stakeholders.

**Results:**

The stakeholders agreed the desired impact was to provide consistent, holistic, patient-centred, and effective EoLC. Four potential long-term outcomes were suggested: (1) increased use of anticipatory and regular end-of-life medications; (2) reduced end-of-life clinical and medication errors; (3) reduced unnecessary hospitalisations; (4) increased concordance between patient preferred and actual place of death. Key interventions focused on providing immediate information on what to do in such situations including: appraising the situation, developing an algorithm for a treatment plan (including whether or not to convey to hospital) and how to identify ongoing support in the community.

**Conclusions:**

A Theory of Change approach was effective at identifying impact, outcomes, and the important features of an end-of-life intervention for paramedics. This study identified the need for paramedics to have immediate access to information and resources to support EoLC, which the workshop stakeholders are now seeking to develop as an intervention.

## Background

Although somewhat contested [1], international evidence still suggests that, for most people, home is their preferred place of death and where they want to be cared for when dying from advanced incurable conditions [2]. The number of people dying at home or in care homes has risen in recent years and is expected to increase in England and Wales by 88.6% and 108.1% respectively by 2040 [3]. Families, friends, and residential care staff therefore play a pivotal role in providing care and support to this patient group, although they can sometimes lack confidence in doing so [4, 5]. At times of uncertainty, often as they witness the worrying and unexpected symptoms of disease deterioration or dying, both informal and formal carers call emergency services for urgent help [6–9]. Consequently, paramedics and the ambulance service can become the patient’s first point of contact in primary care [10]; however, this often leads to more hospital admissions, often against the wishes of the dying person [11, 12]. A review highlighted that only one study reported data on the percentage of end-of life patients transported to hospital following an emergency call [13]. That study found that from 4348 individuals visited by paramedics at the end of life, 74.4% (*n* = 3237) were transported from home, with the most common destination being a hospital (99.5%, *n* = 3221) [14].

Despite the frequent call to ambulance services, and a general recognition that this is a key part of their practice, paramedic confidence in their skills and understanding of the appropriate processes for this particular group of patients is variable [12, 15, 16]. For example, Kirk et al. [17] in their UK survey of 182 paramedics found that 51% reported their end-of-life training as poor, though longer time in service and greater seniority predicted more confidence. Reviews have also highlighted the need for training and information [13, 18, 19]. Moreover, as well as a recognition of training needs, the input and involvement of paramedics to current end-of-life care (EoLC) guidance and policy is lacking [10, p.24].

However, developing interventions to improve practice is challenging, especially when several constraints exist, such as training time and resources. Moreover, previous research has suggested that any intervention implemented needs to be grounded in and informed by paramedic practice for it to be effective [20]. A number of interventions aimed at improving paramedic knowledge around EoLC and reducing unnecessary hospital admissions have been piloted. These include a specialist palliative care telehealth service for paramedics on call-outs [20], the development of a specific protocol [8] and pathway [21], and a package including a palliative care clinical practice guideline, specific training, and mechanism for sharing of goals of care [22]. Additionally Murphy Jones [23] reports on two case studies where comprehensive packages were introduced which targeted a number of areas. While the two interventions described by Murphy Jones [23] differed, key similarities were specialist education for the paramedic workforce and the need for consistency in response.

Understanding the theory underlying an intervention is essential in understanding whether, and how, it works. However, current interventions to improve paramedic practice in EoLC lack a hypothesised theoretical account of how the interventions will lead to the desired effects. The aim of this study was to address this by developing an intervention to improve knowledge and confidence in paramedics when attending dying patients, underpinned by theoretical understanding of how and why the intervention is expected to work in real-world practice.

## Method

### Theory of change approach

One approach to intervention development that specifically addresses the need for theory is Theory of Change (ToC) [24]. Evidence from projects that have used ToC to develop and evaluate complex health care interventions have shown its potential to strengthen all stages of the intervention process, from development to implementation, and to identify the key components and mechanisms of action of complex interventions [24]. ToC does not propose a specific sociological or behaviour change theory but rather provides a framework with which to specify how an intervention affects change [24]. ToC models are becoming increasingly popular in public health interventions [25] and more recently have begun to be used in palliative care [26].

ToC models describe impact (what the intervention aims to achieve), long-term outcomes (the primary outcomes of the evaluation), and pre-conditions (similar to short-term outcomes) [27]. To develop a ToC, stakeholders must work collaboratively to identify how and why they expect an intervention to achieve its intended effect, with the evolving ToC modified throughout the process of intervention development [24, 27]. Causal routes generated by stakeholders to explain how an intervention works can also be visually represented through a ToC map [24]. The ToC is usually co-constructed with participants in a series of workshops using a backwards-mapping approach, starting from identifying the desired impact of the intervention (i.e. what the intervention should achieve), and then working backwards to identify the preconditions (specific outcomes) and intervention activities required to achieve these impacts. During this process, the assumptions about what needs to be in place for the ToC to occur are made explicit.

### Stakeholders

Study stakeholders were healthcare providers with expertise in paramedic EoLC albeit from a number of different perspectives. They included paramedic end-of-life leads, a clinical palliative care consultant, clinical nurse specialists in palliative care, a paramedic pharmacist, and call handler. They were all involved in at least one ToC group workshop (see Table 1).

The number of individuals participating in ToC workshops is influenced by the scale of the intervention and the range of stakeholders who could usefully be involved [28, p.6]. Similarly, no agreement or formal sampling calculation exists on the ideal number of collaborators to involve in the initial workshops to decide on the impact. This study had nine stakeholders participating in the workshops; four service providers participated in the first workshop, five in the second, four in the third, five in the fourth, and six in the fifth. Two key stakeholders attended all the workshops: both paramedics. Each workshop was facilitated by two to three facilitators and lasted between 60 and 90 min; workshops took place approximately two weeks apart.

Recruitment to the workshops was through contacts known to the academic research team and supplemented further by those stakeholders. Participation in the workshops was voluntary, with stakeholders attending as many workshops as they were able. No participation incentives were paid, and all workshops were held online.

**Table 1:** Workshop attendance by collaborators

	Workshop-1	Workshop-2	Workshop-3	Workshop-4	Workshop-5
**Duration**	90 min.	60 min.	70 min.	90 min.	75 min.
**Facilitators**	3	3	3	2	2
**Workshop collaborators (total)**	4	5	4	5	6
Clinical nurse specialist	1	1	1	1	1
Paramedics	2	3	3	2	3
Call handler	1	1	0	0	0
Palliative medicine consultant	0	0	0	1	1
Pharmacist	0	0	0	1	1
**Focus of workshops**					
Intervention impact	X	X	X		
Long-term outcomes		X	X		
Short-term outcomes (preconditions)		X	X		
Intervention activities			X	X	X
Contextual factors (assumptions)	X	X	X	X	X

### Design and procedure

Advice was taken from the academic team’s host institution regarding ethical approval. As all workshop collaborators were considered part of the research team (as opposed to ‘participants’), formal ethical approval was not necessary although ethical principles such as transparency of purpose and voluntary participation were considered important aspects of the ToC generation process. Approval was given from the host service provider for this to be considered a service development project (NWAS_EVAL_0056 approved 3/5/22).

The academic team held five workshops with collaborators between April 2022 and June 2022. The details of attendance and length of each workshop are outlined in Table 1. In accordance with ToC principles, the workshop attendees developed the plans for the interventions in an iterative fashion, starting with identifying the desired impact [24]. The proposed impacts, long-term outcomes, and pre-conditions were all generated from the workshops. In the first workshop, a nominal group technique was used [29]; this is a structured process resulting from small-group discussions to reach consensus by a series of prioritisations. Details of the workshop purpose, discussions, and outputs are provided in Table 2.

**Table 2:** Outline of discussions in each Theory of Change workshop

Workshop	Central theme(s)	Structure	Outputs
1	Deliberated desired impact of intervention. Stakeholders were asked: *“What impact are you looking for in relation to responding to calls where the person is at the end of life?”*	Rapport building Presentations Discussion of paramedic responses to end-of-life calls	Four end-of-life impacts proposed: Holistic, patient-centred, end-of-life care delivered in a timely manner; continuity and consistency of response; unnecessary hospitalisations avoided; improved process of responding to calls
2	Final decision on intervention impact. Stakeholders started identifying long-term outcomes and preconditions.	Discussion and deliberation	Impacts were discussed, reworded, and reduced to two: holistic, patient-centred, and effective end-of-life care; and continuous end-of-life care and consistent response to emergency calls across professions. Initial discussions had on measurable short term outcomes (preconditions) for an intervention.
3	Further discussion to decide appropriate impacts and outcomes. Group began exploring intervention designs.	Discussion and deliberation	Workshop attendees agreed to merge impacts into one: Consistent, holistic, patient-centred, and effective end-of-life care. Discussion produced list of long-term outcomes, such as increase use of medication and reduction in errors, and short term outcomes (preconditions), such as knowledge, identification of end-of-life care patients, and recognising dying. Possible education, communication, and referral based interventions discussed.
4	Intervention development.	Discussions on signs/symptoms of dying and key elements for care of the patient in paramedic practice Presentation and demonstration of existing tools for assessing need.	Limitations and strengths of existing tools and resources identified. Identified need to develop an online training and/or a prompt card with QR codes to signpost to relevant material. Identified need for public facing information resource.
5	Finalising intervention	Presentation of evidence from literature Discussion of intervention components.	Developed ideas for decision-making algorithm. Agreed that: intervention should be incorporated into information sources used by paramedics; identifying a patient as being at the end of life is key and should be the focus, followed up with further guidance; foundational knowledge is important; prompt card is good idea; public information leaflet not suitable for this project given time, resource, and focus.

In between workshops, the core research team (NP, BR, TB, KP, JS) analysed the workshop discussion, considered what intervention activities would be possible within the limited time and funding resources, and generated a ToC map.

### Data analysis

**Fig. 1:**
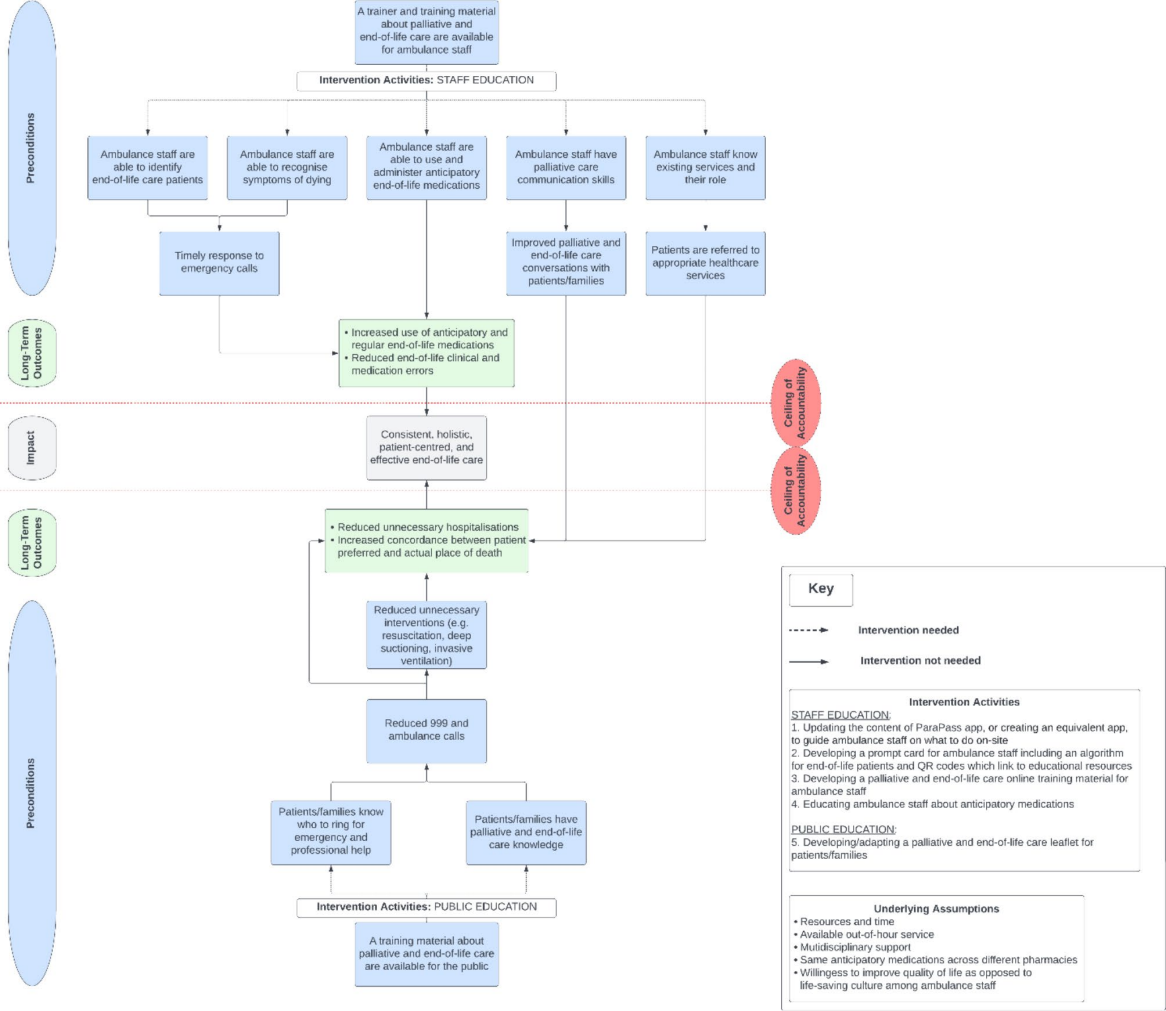
ToC map

Data analysis occurred concurrently with data collection and was the basis for feedback throughout the workshops through sharing the emerging components of the ToC map (Fig. 1). During the workshops, the map was built in a stepwise fashion, beginning with determining the impact of the intervention and ending with discussing what was needed to achieve the impact given the available resources and contextual elements in their daily practice. The wider literature on end-of-life practice in paramedic care was reviewed to provide background information, guide focused discussions with the stakeholders, aid in constructing the ToC map, and support the validity of the causal links in the hypothetical pathway of change.

## Results

There was consensus between stakeholders with regards to the important impacts and outcomes of the intervention, although with some difference in perspectives on how extensive the scope of the intervention should be. Stakeholders ultimately agreed the desired impact of an intervention is to ensure paramedics can provide consistent, holistic, patient-centred, and effective EoLC. The proposed long-term outcomes included increased use of end-of-life medications, reduction of errors, reduction of unnecessary hospitalisations, and increased concordance between patient preferred and actual place of death. Fourteen preconditions were proposed, including increasing ambulance staff’s knowledge and reducing unnecessary interventions. The relationships between outcomes, underpinning assumptions, and hypothetical pathway of change are depicted in the ToC map (Fig. 1). The outputs of each ToC workshop are summarised in Table 2.

### Assumptions

Workshop collaborators discussed contextual factors that could influence the effectiveness of the proposed interventions in each workshop. They agreed on five key assumptions: services have sufficient resources and time for the intervention; out-of-hours support is available; there is multi-disciplinary support; the same anticipatory medicines are available in different pharmacies; and a willingness to improve quality of life as opposed to a life-saving culture among ambulance staff.

### Describing the components of the proposed intervention

While multiple different intervention types were discussed, workshop collaborators and the research team identified the need for paramedics to have immediate access to information resources through the systems already in use when out on call. Because of limited time and funding within the paramedic service to develop the intervention, it was decided to proceed with the development of:

- An online end-of-life resource for ambulance staff. This would entail redesigning an existing app with a decision-making tool, short video resources, and signposting information. This would serve as a brief resource for paramedics to use on site at the point of need, integrated into multiple existing systems for supporting paramedic decision-making. The workshop collaborators would be involved in refining the online resource throughout development.
- A prompt card for staff with QR links. This could be an alternative source of basic information for paramedics attending calls in areas of poor internet access. It could include some prompts about decision-making for end-of-life patients and a QR link to the resources described above, internet connection allowing.

## Discussion

The aim of this study was to use a ToC approach to develop an intervention to improve paramedic professionals’ knowledge and confidence when attending an individual at the end of life. Over several workshops, stakeholders agreed the intended impact of such an intervention was for paramedics to provide consistent, holistic, patient-centred, and effective EoLC. A proposed intervention was for an online end-of-life resource and a prompt card, both of which are integrated into existing systems and processes, and can be immediately accessed by staff attending a patient. Suggested long-term outcomes included increased use of end-of-life medications, reduction of errors, reduction of unnecessary hospitalisations, and increased concordance between patient preferred and actual place of death. To achieve these outcomes, 14 preconditions were proposed, including increasing ambulance staff’s knowledge of relevant issues and reducing unnecessary interventions and emergency calls.

The stakeholders involved in this study identified the need for more training, increased knowledge, and education about palliative care among paramedics, reiterating findings from other studies [13, 18]. Given some of the similarities between intentional concerns and the desired outcomes identified in this study, the ToC specified in this study may have clinical utility outside the UK. Where this study differs somewhat is that it identified the importance of immediate access to online and other resources, rather than a focus on higher system-level interventions described elsewhere [15, 22]. The involvement of staff, including ‘frontline’ paramedics, has likely shaped the immediate and practical – rather than systemic – focus of the interventions. This is arguably a strength of the study, as stakeholders identified a low cost and feasible intervention, with few demands on either time or financial resources for implementation.

While acknowledging the practical focus of the intervention, supported by the ToC approach focusing on feasible short and long-term outcomes, the workshop attendees agreed that some contextual factors need to be in place for the intervention to work. Some of these, for example having resources and time, may be difficult to achieve in the context of overburdened health and social care sectors. Wider systemic issues referenced in other literature should also not be neglected. For example, the individualised approach to paramedic decision-making at the end of life highlighted in other studies calls for better access to community care and improved information sharing across services [7]. Further work developing an intervention would have to aim to ensure, for example, it is feasible for paramedics to commit time and resource to this. Nonetheless, paramedic services are limited in the impact they can have on these wider issues and part of an intervention based on this ToC would involve examining the possible impact and effectiveness within different contexts, thereby assessing the importance of different assumptions. Additionally, paramedic services looking for ways to improve care of patients at the end of life will benefit from a low-cost intervention that can be easily implemented, evaluated, and built upon. Informal feedback from workshop attendees in this study suggested that they found the experience useful and constructive, with an expressed wish to continue through the later stages of the project including full intervention development and implementation.

The next stage of the research is to operationalise and evaluate the different elements of the intervention. One of the benefits of the ToC approach is to help guide the evaluation of the intervention by assessing/measuring each outcome of the proposed mechanism of change. For example, it is possible to assess whether (1) ambulance staff will be able, after the intervention, to identify accurately EoLC patients, (2) which would increase timely response to emergency calls, (3) which, in turn, would help increase the use of anticipatory and regular end-of-life medications and reduce drug errors (4) which would deliver the overall expected impact. If the anticipated long-term outcomes have been achieved, all these individual theoretical components can be assessed to confirm the theoretical path. If the long-term outcomes are not achieved, the assessment of individual components can help make sense of where the intervention has not been successful or where the causal chain has broken down.

## Conclusions

The five group workshops, supported by the research team discussions and knowledge of relevant literature, enabled the production of a ToC model. The agreed intervention impact was to provide consistent, holistic, patient-centred, and effective EoLC. Stakeholders proposed key long- and short-term outcomes for an intervention to achieve that impact, including increasing staff knowledge and increase use of end-of-life medications. The stakeholders in this study highlighted the importance of paramedics having immediate access to information to support end-of-life decision-making and difficult conversations, backed up by palliative care training and education of the workforce.

## Data Availability

The data from this study have been included in the manuscript.
